# When advance directives clash with family consent: designing an operational framework for end-of-life decision-making in China and Korea

**DOI:** 10.1186/s12910-026-01451-1

**Published:** 2026-04-01

**Authors:** Songwu Luo, Dongje Cho, Jaehyun Cho

**Affiliations:** Department of Law, Dong-A University, Bumin Campus, 225, Gudeok-ro, Seo-gu, Busan, 49236 Republic of Korea

**Keywords:** advance directives, life-sustaining treatment, surrogate hierarchy, safe harbor, duty to search, comparative law, China, Korea

## Abstract

**Background:**

End-of-life decisions in East Asia often juxtapose written advance directives (ADs) with family-centered consent, generating recurrent conflicts at the bedside. While scholarship richly describes cultural rationales, the operational translation of legal-ethical principles into implementable hospital procedures remains underdeveloped. This study addresses that gap by proposing a comparative, workflow-ready blueprint that integrates priority rules, a duty-to-search protocol, and clinician safe-harbor protections across China (PRC; mainland China) and the Republic of Korea (South Korea) frameworks.

**Methods:**

We conducted systematic doctrinal analysis of Chinese and Korean legal frameworks, followed by normative design of operational tools. Framework coherence was evaluated through structured scenario analysis of 12 literature-derived cases using established walkthrough methodology—a non-empirical validation approach standard in policy design.

**Results:**

We present three integrated tools: (1) the Autonomy-Prioritized Decision Model (APDM; the “3 + 1” structure—three decision tiers plus one execution step) establishing decision hierarchy (Layer 1: Verifiable AD; Layer 2: Appointed Proxy; Layer 3: Default Kin; +1: Order Execution); (2) an auditable “Duty-to-Search” (DtS) protocol converting vague obligations into time-bound, documentable actions with mandatory EHR fields; and (3) dual-model Safe-Harbor clauses linking procedural compliance to liability protection. Scenario-based walkthroughs across 12 conflict types achieved 100% decision-path convergence after iterative refinement (initial convergence: 83%), with 75% appropriately triggering ethics consultation, demonstrating logical coherence and operability across three legal environments (Korea, CN-Local, CN-No-Rule) via jurisdiction settings.

**Conclusions:**

By converting autonomy and relational concerns into operational law, the blueprint supplies regulators and hospitals with actionable design patterns to reconcile ADs with family practices in China and Korea. It is portable to other East Asian jurisdictions, supports incremental adoption through local bylaws and hospital policies, and delineates measurable compliance tools for quality assurance. This approach provides a basis for future mixed-methods evaluation.

**Supplementary Information:**

The online version contains supplementary material available at 10.1186/s12910-026-01451-1.

## Background

### The normative conflict and its consequences

End-of-life (EOL) care in East Asia repeatedly confronts a structural bedside conflict: a patient’s documented wishes in an advance directive (AD) may be contradicted by contemporaneous family demands. This is not merely interpersonal disagreement but a normative collision between individual self-determination and family-centered decision practices, producing a high-stakes risk triangle for patients, clinicians, and institutions [1]. For patients, the conflict can translate into over- or undertreatment that defeats advance planning; for clinicians, it generates moral distress and legal uncertainty; and for institutions, it can lead to inconsistent or ad hoc resolution pathways—most prominently in China given the absence of a nationally codified enforceability regime for ADs, and in South Korea primarily in time-pressured or edge-case settings where implementation can vary across clinical contexts despite a codified statutory pathway. These recurring conflicts motivate the need for a workflow-ready, legally traceable protocol that preserves the patient’s verifiable voice while structuring family involvement through auditable steps and escalation pathways.

### Legal-ethical landscape in china and korea

Legal baseline (why family consent matters, and when it does not). In both jurisdictions, family involvement is practically salient, but its legal role differs sharply. In South Korea, the LSTDA creates a codified pathway: when a legally compliant AD (or equivalent registry-verified instrument) is established, clinicians are expected to follow it as the controlling expression of the patient’s will, while family members participate primarily through communication and dispute mediation channels. Family decision-making becomes legally operative only when no controlling directive exists, and then only within the statute’s surrogate hierarchy and procedural safeguards (including institutional ethics review).

In China, the Civil Code supplies autonomy-relevant principles but does not itself provide a nationwide enforceability regime for ADs; accordingly, family/guardian-based consent often functions as the default proxy mechanism in incapacitated care. At the same time, local enabling rules (e.g., Shenzhen) can make certain documented patient instructions operationally enforceable, requiring medical institutions to respect a clear written/audiovisual instruction annotated in the medical record.

Implication for this paper. Our model is designed to be law-consistent by construction: in Korea (and in Chinese local-enabled settings), it does not authorize overriding a verifiable directive based on family preference; in “no-rule” settings, it treats ADs as strong evidence and routes high-risk conflicts through mandatory ethics escalation before withdrawal decisions.

In China, the national framework for ADs is principles-based rather than codified [2], with enforceable local breakthroughs. The Civil Code of the People’s Republic of China (2021) provides a crucial, albeit general, foundation. Its Part on Personality Rights upholds bodily integrity, and Book VII (Tort Liability) contains provisions on medical informed consent (e.g., Article 1219). Together, these support autonomous healthcare decision-making but do not establish nationwide validity, formal requirements, or enforceability for ADs. Practical enforceability currently hinges on pioneering local legislation. The leading example is the Shenzhen Special Economic Zone Medical Regulations (revised 2022), whose Article 78 requires that, where a capacitated patient has made a clear expression in written or audiovisual form and the instruction is annotated in the medical record, medical institutions must respect that wish.

In contrast, South Korea has adopted a centralized and highly codified national approach [3]. The Act on Decisions on Life-Sustaining Treatment for Patients at the End-of-Life (LSTDA), in force since 2018, together with the Enforcement Decree and Enforcement Rule, establishes a comprehensive framework. It defines types of life-sustaining treatments, mandates statutory forms for ADs and Physician Orders for Life-Sustaining Treatment (POLST), establishes a national registry, and specifies a surrogate hierarchy. Hospital Ethics Committees (HECs) are mandated to mediate disputes and verify processes. While this system offers clarity, its formalities and procedures can themselves create delay and conflict in fast-paced settings.

In this article, “China” refers to the People’s Republic of China (PRC; mainland China). We discuss Taiwan only as a distinct legal jurisdiction when referencing its advance care planning framework for comparative purposes; this terminology is used for analytical clarity and does not express any position on sovereignty or international legal status.

### Literature gap and study aims

International frameworks provide valuable comparative insights [4]. The UK’s Mental Capacity Act (2005) establishes ‘best interests’ standards with clear statutory guidance [5]. Singapore’s Advance Medical Directive Act demonstrates Asian adaptation of Western models [6], part of broader regional efforts to develop culturally-appropriate frameworks [7]. Japan’s experience with culturally-tailored advance care planning offers particularly relevant implementation insights [8]. Germany’s patient decree (Patientenverfügung) system offers lessons on binding directives within civil law traditions [9].

The literature richly explores cultural attitudes (e.g., filial piety, relational autonomy) and policy/ethics comparisons [10], with systematic reviews identifying multiple implementation barriers [11], yet it lacks operational research translating principles into granular clinical workflows. This study develops an integrated, executable model to resolve AD–family conflicts in China and Korea by: (1) designing an Autonomy-Prioritized Decision Model (APDM) with an explicit hierarchy of decision authority; (2) defining operational duties and protections via auditable checklists and safe-harbor clauses; and (3) providing implementation-ready tools (flowcharts and model clauses). We target the “last mile”: converting autonomy into a verifiable, auditable, and legally robust sequence of bedside actions.

### Normative foundations: reconciling autonomy and family involvement

The perceived conflict between Western individualism and East Asian familialism has long shaped end-of-life discourse [12], and recent studies continue to explore this tension in both domestic and diaspora contexts [13–15]. We reject this false dichotomy by treating autonomy as relationally embedded: family involvement often supplies informational and emotional scaffolding for patient choice, but that scaffolding does not automatically translate into a veto over verifiable patient preferences. This stance aligns with contemporary accounts of relational autonomy that emphasize supportive relationships and communicative conditions without collapsing autonomy into family preference [16]. It also provides a clearer normative basis for designing workflows that engage families while keeping the patient’s expressed values as the organizing reference point.

We operationalize Mackenzie & Stoljar’s (2000) relational autonomy [17] by translating theory into bedside decision rules. Subsequent work in bioethics and medical law has further refined relational autonomy for clinical contexts, clarifying how clinicians can respect the patient’s will while navigating family involvement [18]. Our layered model provides the “operational specificity” needed for consistent application: Layer 1 preserves the individual voice when a directive is verifiable and applicable; Layer 2 formalizes trust through an appointed proxy bounded by substituted judgment; and Layer 3 defaults to kin-based surrogacy only when the patient’s own verifiable preferences are unavailable. These design choices are consistent with legal and ethical analyses of surrogate authority and family roles in medical decision-making [19, 20].

A common critique is that directive primacy imposes “Western” values onto East Asian settings [21]. We counter that honoring verifiable directives often protects the relational processes that produced them, while procedural safeguards are necessary because surrogates can misjudge patient wishes under grief, guilt, and social pressure. Accordingly, the framework gives families meaningful roles through required communication touchpoints and grants them full decision authority when no directive exists, while preventing distortions that contradict patient values through escalation and documentation. The safe-harbor component does not enable clinicians to bypass families; it protects clinicians who follow required communication steps and ethics-mediated resolution in high-conflict cases, and it can be supported in practice through multidisciplinary ethics support structures [22].

## Methods

This study adopts a three-stage methodology that translates legal–ethical principles into an operational bedside workflow. We explicitly take a normative design approach to produce a proof-of-concept framework rather than an empirically validated clinical tool. No human participants or identifiable clinical data were involved; ethics approval was therefore not applicable. The method integrates: (i) doctrinal and comparative legal analysis to establish the “law-in-the-books” baseline and surface operational gaps; (ii) a structured normative design methodology that engineers auditable tools under real-world constraints; and (iii) an analytic scenario-based validation to exercise the tools conceptually, identify failure modes, and refine decision logic. This study establishes theoretical operability, legal traceability, and ethical defensibility as the foundation for future empirical validation.

Plain-language terminology. To improve readability, we use plain clinical language where possible. Throughout the paper, we use “tools” (or “protocol tools”) to refer to what design-science literature often calls “artifacts”. We use “decision pathway” (or “decision logic”) to refer to the core set of rules that determine whose decision governs and what steps follow. These wording choices do not change the substance of the framework; they only make the presentation easier to follow.

### Doctrinal and comparative legal analysis

Our foundational layer is a systematic doctrinal analysis of primary legal sources governing end-of-life (EOL) decision-making in the target jurisdictions. For China, we analyzed the Civil Code’s Personality Rights Book and tort/liability provisions to map the principles-level framework structuring patient autonomy, informed consent, and bodily integrity. We examined representative local regulatory practices, with Shenzhen’s rules as the leading reference for “CN-Local,” to understand how advance directives (ADs) and surrogate decisions are operationalized at facility level. For Korea, we analyzed the Life-Sustaining Treatment Decisions Act (LSTDA) with its Enforcement Decree and Rule, including statutory forms (Form No. 6 for ADs; Form No. 13 for POLST).

The doctrinal analysis is complemented by comparative functional review using the UK Mental Capacity Act 2005 (for “best interests” determinations and “reasonable steps” doctrine) and Taiwan’s Patient Right to Autonomy Act 2019 (for advance care planning mechanisms and registry standards). We selected these two jurisdictions purposively as complementary functional exemplars rather than as exhaustive comparators. The UK framework provides a mature and widely cited statutory articulation of “best interests” reasoning and clinician due-diligence standards (e.g., “reasonable steps”), while Taiwan offers an East Asian, culturally proximate model that operationalizes advance care planning through structured procedures and registry-linked implementation; together they supply mechanisms directly relevant to our design goals. Rather than descriptive taxonomy, we pursued functional importation: identifying mechanisms with demonstrated clarity elsewhere and assessing their transposability to China and Korea. This paired analysis established: (a) lawful documentation standards, (b) decision rights allocation among directives, proxies, and relatives, and (c) operational gaps preventing reliable bedside execution.

### Normative design methodology

We framed the AD–family conflict as a normative design problem bounded by multiple real-world constraints. In acute-care contexts—particularly the ED and ICU—clinical time pressures often demand rapid decision-making, leading us to establish policy targets of 30–60 min for initial ED searches and 2 h for comprehensive ward searches—configurable default windows for implementation and auditability—normative benchmarks for workflow design and auditing rather than empirically observed performance outcomes. In non-urgent settings (e.g., planned admissions, routine inpatient care, or long-term care), the same workflow can be applied with appropriately extended timelines and deliberation windows. The legal heterogeneity between Korea’s codified system (statutory forms, national registry) and China’s variable landscape (principles-based national law with local enabling rules or none) necessitated a jurisdictional toggle mechanism supporting three modes: KR (statutory verification), CN-Local (local rule application), and CN-No-Rule (directive as primary evidence with ethics consultation). Ethical considerations required balancing patient autonomy with relational values while providing fair-notice protections for clinicians through transparent protocols [23].

Based on these constraints, we developed four interlocking tools: (1) the Autonomy-Prioritized Decision Model (APDM; “3 + 1” structure) establishing decision hierarchy across verifiable directives, appointed proxies, and default kin, plus execution layer; (2) a Duty-to-Search protocol converting vague obligations into auditable, time-bound actions with mandatory EHR fields; (3) safe-harbor model clauses linking procedural compliance to liability protection; and (4) workflow charts with EHR integration schemas enabling jurisdictional adaptation. Full specifications appear in Results; here we note that each artifact addresses specific operational gaps identified in our legal analysis.

Iterative refinement. We treated this four-artifact set as Version 1 (v1) and then exercised it through the scenario-based walkthrough (Sect.  3.3). Where the walkthrough exposed a failure mode—most notably divergent judgments about whether a discovered directive was applicable to the immediate intervention—we revised the APDM by embedding a brief “Applicability Checklist” within Layer 1 to standardize scope parsing and to trigger ethics escalation when contested (Supplementary Table S5). Accordingly, the final framework retains the same four core tools, with the Checklist functioning as an internal refinement module of the APDM rather than an additional standalone tool.

Terminology note: We use AD to refer to patient-authored advance directives generally. We use POLST functionally to refer to executable physician orders or their statutory implementation equivalents across jurisdictions. In Korea, this does not correspond to a single identically named POLST form, but is operationalized through the statutory patient-intent confirmation and implementation pathway under the LSTDA framework, including the implementation form (Form No. 13). We use HEC consultation as an umbrella term for hospital-based ethics consultation mechanisms (HEC or MDT-based ethics support, depending on institutional capacity and jurisdiction).

### Scenario-based validation

Scenario-based validation, also known as ‘walkthrough evaluation,’ is an established design validation method in healthcare informatics and policy design [24]. This method is particularly valuable for testing decision algorithms and workflows before implementation, as demonstrated in clinical decision support systems and emergency protocols [25]. We emphasize that this validation serves as theoretical testing of internal logic and consistency, not empirical validation of clinical effectiveness. Scenario-based walkthrough is an established method in policy and systems design, particularly useful for testing decision logic without human subjects [26].

We selected 12 scenarios based on a 3 × 4 matrix design to ensure systematic coverage. The scenario development followed a structured literature-based approach. First, we systematically reviewed case reports and clinical ethics literature from major databases (PubMed, Web of Science, CNKI) spanning 2020–2024, using search terms including ‘AD conflict,’ ‘family disagreement end-of-life,’ and ‘surrogate decision dispute’ in both English and Chinese. This yielded 47 documented conflict cases. We then categorized these cases by clinical setting (ED/ICU/Ward) and conflict type (AD vs. family, proxy vs. family, etc.), selecting the most representative scenario for each matrix cell based on: (1) frequency of occurrence in literature (appearing in ≥ 3 independent sources), (2) jurisdictional relevance (documented in Chinese or Korean contexts), and (3) decision complexity (requiring framework navigation). This number (12) represents the minimum set needed to test all critical decision pathways while remaining manageable for detailed walkthrough analysis. This structured search was used for scenario construction and selection (i.e., literature-informed sampling) rather than to produce a systematic review dataset. The distribution ensures each clinical setting (ED, ICU, ward) and conflict type is tested under multiple jurisdictional conditions (KR, CN-Local, CN-No-Rule).

Scenarios were developed through an iterative process. Initial scenarios derived from published case reports and clinical ethics literature were refined through consultation with the research team’s clinical member to ensure plausibility. Each scenario specified: patient background (age, diagnosis, capacity), jurisdictional setting, information availability (document presence/format, registry accessibility), and stakeholder positions (patient values, proxy stance, relatives’ views).

Two researchers—one with legal/regulatory expertise, one with clinical operations experience—independently walked each scenario through the framework using structured prompts: Which search steps trigger? Which EHR fields require completion? Which priority rules apply? What orders result? Are ethics consults required? Both walkthrough evaluators were members of the author team. Accordingly, this walkthrough was designed as an internal validation of logical consistency and jurisdictional compliance, rather than an external test of real-world usability under time pressure in clinical settings.

We compared decision paths to assess consistency and identify divergence points. Here, “Round 1” refers to the independent walkthrough of all 12 scenarios using the initial (v1) framework artifacts. We defined “path convergence” as agreement on (i) the active jurisdictional toggle (KR/CN-Local/CN-No-Rule), (ii) the APDM layer selection, (iii) whether an ethics trigger was required, and (iv) the resulting executable order/output (+ 1 translation). “Refinement” denotes targeted adjustments to the decision logic prompted by documented divergence points (e.g., applicability parsing), after which all scenarios were rerun in “Round 2” to confirm stable convergence. The ≥ 10/12 convergence threshold was selected as it allows minor interpretive differences while ensuring substantial agreement. This 83% initial agreement rate is comparable to inter-rater reliability standards in qualitative research, though we emphasize this is for design consistency, not statistical validation. Divergences prompted targeted refinements until achieving 100% convergence across two consecutive rounds, confirming internal coherence without dead-ends or jurisdiction-rule violations. Specifically, in the first walkthrough round, two scenarios diverged due to directive-applicability ambiguity (e.g., whether declining “mechanical ventilation” includes short-term post-operative ventilation, and whether “no prolonged suffering” encompasses PEG placement). We addressed this by adding the three-item Applicability Checklist (AC-1 to AC-3) as a Layer 1 decision gate and reran the walkthrough; the second round achieved full convergence.

### Ethical and legal justification analysis

For each design decision, we conducted paired justification analysis. Legal justification linked operational steps to specific legal hooks: KR verification through LSTDA forms/registry; CN-Local recognition via enabling rules; CN-No-Rule treatment of directives as primary evidence requiring ethics consultation. Safe-harbor templates align with civil-liability architectures through rebuttable presumptions tied to documented compliance.

Ethical justification grounded the framework in respect for autonomy while operationalizing relational autonomy—family input is solicited but cannot negate verifiable directives. Non-maleficence and beneficence are served through emergency overrides and time-bounded searches. Justice is advanced through standardized documentation reducing arbitrary variation. The duty-to-search converts moral aspiration into auditable conduct.

### Study limitations

This normative design study produces a proof-of-concept framework with several limitations. First, this normative design establishes the framework architecture; clinician uptake, real-world time costs, and acceptability will be evaluated in subsequent empirical studies. In particular, because the scenario-based walkthrough was conducted by the study authors in a desk-based setting, it primarily demonstrates internal coherence of the decision logic and does not establish real-world usability (e.g., learnability, cognitive load, speed, or error rates) for busy emergency clinicians. Second, synthetic scenarios cannot capture full clinical variability; agreement thresholds function as design gates, not reliability evidence. Third, legal mapping reflects sources through September 2025; subsequent changes may alter recognition pathways. Fourth, generalizability outside East Asia remains untested.

Future mixed-methods pilots will evaluate process metrics (time-to-decision, search completeness), fidelity metrics (target threshold achievement), and acceptability through stakeholder interviews. The method intentionally separates normative engineering from empirical evaluation, delivering a legally traceable, ethically reasoned framework ready for IRB-approved piloting without claiming current real-world effectiveness.

### Study scope and boundaries

We explicitly position this as a normative design study—developing theoretically grounded, legally compliant frameworks without empirical validation. This approach, common in legal scholarship and policy design [27], allows rigorous development of implementation blueprints before resource-intensive pilots. The absence of stakeholder consultation, while limiting face validity, ensures the framework’s logical integrity is not compromised by institutional politics or professional interests that often dilute reform efforts. The framework assumes no general legal or ethical basis to override a clear, applicable AD for social-utility reasons. Rare mandatory-treatment contexts driven by public law (if any) are outside the scope of this bedside AD–family conflict workflow and would require separate legal authorization. Accordingly, exceptional measures in this workflow are permitted only as auditable, time-limited, and reviewable steps designed to manage verification uncertainty or imminent instability—not as open-ended discretion to deviate from a clear, applicable directive.

## Results

**Fig. 1:**
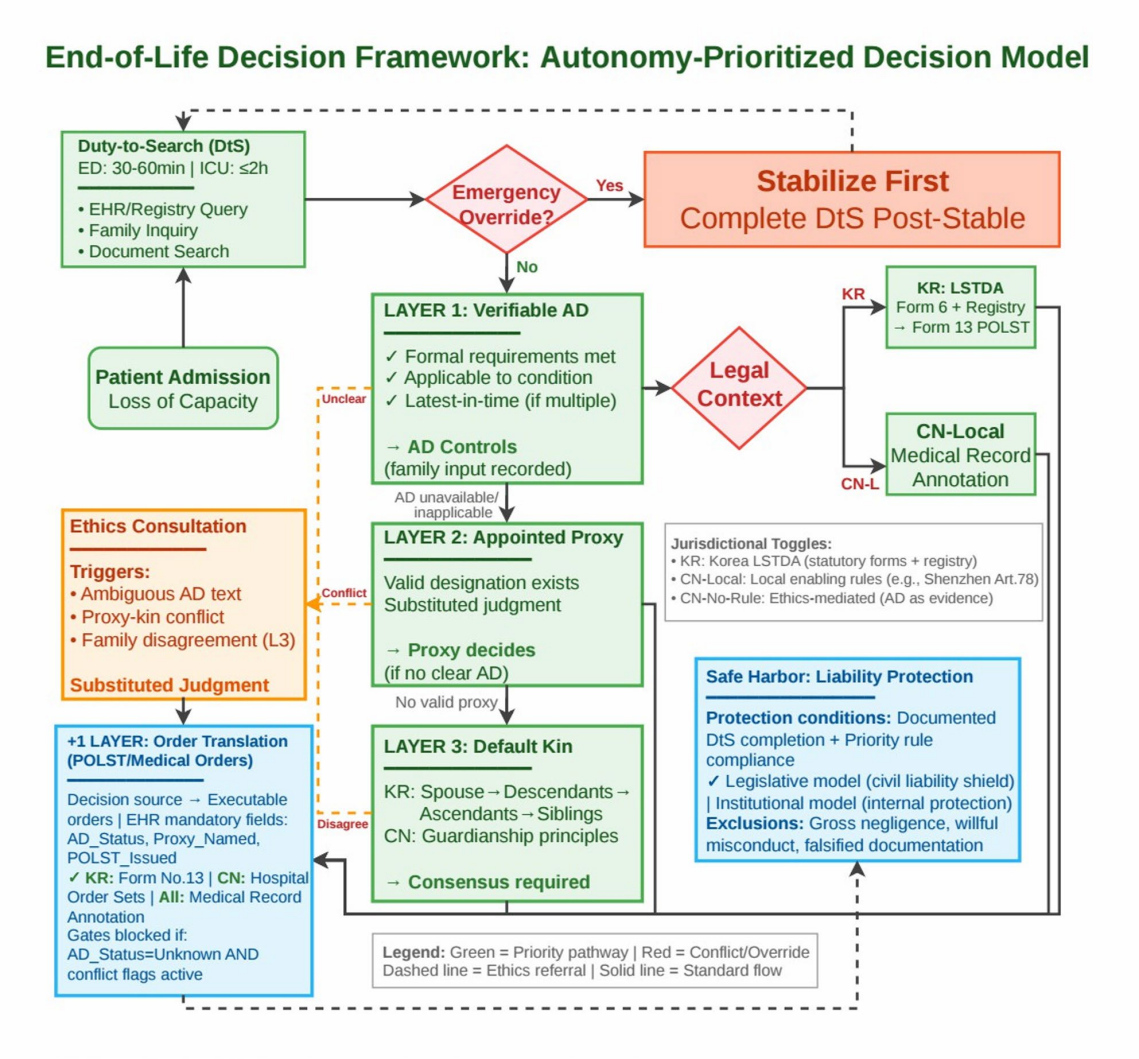
Autonomy-Prioritized Decision Model (APDM; the “3 + 1” structure) and bedside conflict workflow (ED/ICU/Ward). Legend includes Latest-in-Time, Directive-vs-Proxy, and Emergency Override; CN branches show Local-enabled vs. No-Rule paths. Note: Figure 1 depicts the core decision pathway of the framework. It answers the bedside question “whose decision governs, when, and how?” by sequencing the analysis through three decision layers and one execution layer, each bound to concrete checks and documentation outputs. Emergency Override is a time-limited stabilization gate triggered only under predefined criteria; it does not displace the Layer 1–3 hierarchy once verifiability/applicability is established. Supplementary Figure S1 provides the full step-by-step logic. Operative EHR fields for DtS auditability are listed in Supplementary Table S2, and complete safe-harbor model clauses are provided in Supplementary Text S1

Our normative design approach yielded an operational decision framework integrating four core tools: (i) a comparative legal foundation with jurisdiction settings (Table 1), (ii) the Autonomy-Prioritized Decision Model for decision precedence (Fig. 1), (iii) an auditable Duty-to-Search protocol (Table 2), and (iv) safe-harbor provisions for clinician protection. These tools form a coherent bedside pathway internally validated for logical consistency through systematic scenario testing.

### Comparative legal foundation

Table 1 synthesizes the operational differences between Chinese and Korean legal frameworks, revealing a fundamental divergence that shapes our design. Korea’s LSTDA provides high procedural clarity through statutory forms (Form No. 6) and a national registry [28], offering legal certainty but potentially excluding authentic wishes failing strict requirements [29]. Conversely, China’s Civil Code provides only high-level principles without national surrogate hierarchy or AD enforcement mechanisms, creating ambiguity but enabling local innovation like Shenzhen’s Article 78, which grants enforceability to written/audiovisual directives with medical record annotation.

**Table 1:** Core Operational Elements of End-of-Life Decision-Making Frameworks

Legal Element	China (Civil Code & Local Regulations)	South Korea (LSTDA & Implementing Rules)
Legal Status of AD	No unified national statute; principles-level framework; enforceability relies on local regulations.	Formally recognized and legally binding if compliant with statutory form.
Formal Requirements	National: general principles; Local: written/audiovisual, clear expression, medical record annotation.	Statutory forms; consultation and registration required.
Default Surrogate Order	No specific national hierarchy; relies on guardianship principles; local/hospital policy may specify.	Explicit hierarchy: Spouse → Lineal descendants → Lineal ascendants → Siblings; intra-tier consensus required.
Dispute Resolution	No national dedicated mechanism; civil litigation or local ethics committees if mandated.	Institutional HECs mandated; courts as last resort.
Safe Harbor for Clinicians	No explicit national provision; contingent on adoption in local law/bylaws.	Compliance with LSTDA procedures affords strong defense (not labeled “safe harbor”).

Legal sources include China’s Civil Code (2021) and local regulations (e.g., Shenzhen Medical Regulations Art. 78), and South Korea’s LSTDA (2018) with implementing rules. Full legal citations in Supplementary Table S1.)

This divergence necessitates jurisdiction settings allowing the framework to operate across three contexts: KR (Korea’s codified pathway), CN-Local (China with enabling rules like Shenzhen), and CN-No-Rule (China without specific local legislation). The framework adapts its verification requirements, documentation standards, and escalation pathways based on the active jurisdiction, ensuring legal compliance while maintaining operational consistency.

### Toggle definitions: verifiable AD, surrogate authority, and dispute pathways

KR (lex lata; LSTDA mode). Verifiable AD means a registry-verified statutory instrument (e.g., LSTDA-recognized forms) that is applicable to the present clinical decision and not revoked. Surrogate authority applies only in the absence of a controlling instrument and follows the LSTDA hierarchy (including intra-tier consensus where required). Dispute pathway is routed through the institutional Hospital Ethics Committee (HEC) process specified under the LSTDA framework, with courts as a last resort.

CN-Local (lex lata exemplar; local enabling mode). Verifiable AD refers to a clear written or audio/visual instruction that is annotated in the medical record and recognized as operationally effective under an enabling local rule (e.g., Shenzhen-style regimes). Surrogate authority otherwise defaults to guardianship/family-consent principles as operationalized by the facility’s policy in the absence of a controlling, locally recognized directive. Dispute pathway is not standardized nationally; where available, institutions may use ethics committee review and documented mediation, while external civil/administrative channels remain possible.

CN-No-Rule (lex lata; non-enabling mode). There is no nationwide Chinese enforceability regime that renders ADs uniformly controlling. Accordingly, verifiable AD is treated as graded evidence of patient values and preferences rather than an automatically controlling order. Surrogate authority in practice defaults to guardianship/family-consent mechanisms in incapacitated care. Dispute pathway is likewise not nationally standardized; in this mode, the framework’s mandatory ethics escalation and auditable documentation are presented as a policy/implementation safeguard (not a statement of nationwide legal effect) to reduce clinician exposure in high-risk conflicts.

For implementation, we publish a consolidated if–then rule set that operationalizes the DtS protocol, jurisdiction settings (KR/CN-Local/CN-No-Rule), APDM layer selection, and + 1 order translation; see Supplementary Table S6.

### Autonomy-prioritized decision model

Figure 1 presents the core decision pathway that answers “whose decision governs, when, and how?”

Capacity-first principle. This framework is primarily designed for situations in which the patient lacks decision-making capacity. When a patient is conscious and has decision-making capacity, the patient’s contemporaneous informed decision governs and may revoke or modify any prior AD; such updates should be documented in the medical record (and, where a registry exists, updated accordingly).

Emergency Override (time-limited stabilization gate). In this framework, “Emergency Override” does not mean disregarding patient autonomy. It denotes a narrow, time-limited stabilization exception used only when imminent physiologic instability makes delay likely to cause death or irreversible harm and the directive/order cannot yet be rapidly verified or its applicability is genuinely uncertain. Operationally, the “override = yes/no” outcome is determined by a gated decision rule, not assumed as a default first step. The gate is triggered only when all of the following conditions are met: (EO-1) imminent life-threatening instability; (EO-2) no immediately verifiable, applicable directive/order at hand; and (EO-3) the intervention is necessary and reasonably reversible to create time for verification, documentation, and (if needed) ethics escalation. Once the patient is stabilized—or once the directive/order becomes verifiable and applicable—orders must revert to the governing authority identified by Layers 1–3, with documentation of the trigger, duration, and post-stabilization alignment. For avoidance of doubt, this gate is not a mechanism to override a verifiable, applicable AD based on third-party welfare (e.g., perceived family or societal need). Once a directive is verifiable and applicable, it governs unless the patient has capacity and contemporaneously revokes/updates it or the directive is legally invalid/non-applicable.

Safeguards against misuse. The framework treats “extraordinary decisions” as gated exceptions, not discretionary departures. First, Emergency Override is triggered only under explicit criteria (EO-1 to EO-3) and is time-limited, expiring once stabilization is achieved or once the directive/order becomes verifiable and applicable. Second, any exceptional step must be documented in an auditable manner (trigger rationale, time window, actions taken, and post-stabilization alignment), which enables retrospective review. Third, when disagreement persists or when the exception materially alters the care trajectory (e.g., time-limited trials or contested applicability), the pathway requires ethics consultation/HEC escalation and records the justification for the resulting order set. Finally, the safe-harbor logic is procedurally conditional: clinician protections attach to adherence to these steps, creating an incentive to follow the workflow rather than bypass it.

### Layer 1 — verifiable advance directive (highest priority)

Entry criteria. Layer 1 applies when an advance directive (AD) is verifiable and operative under the active jurisdictional mode. In Korea, verifiability requires a registry match (and the relevant statutory form where applicable). In CN-Local settings, verifiability requires a documented directive that is annotated in the medical record in accordance with local enabling rules. In all modes, the directive must be applicable to the present clinical condition and not revoked; where multiple directives exist, the latest-in-time verifiable directive governs. If the patient has capacity and expresses a different preference, that contemporaneous statement is treated as the latest-in-time revocation/update and is documented before any order translation.

Justification. This prioritization reflects the legal-ethical commitment to patient self-determination and, where binding rules exist (KR and CN-Local), aligns clinical action with the governing legal pathway for recognizing patient instructions.

Family role and ethics triggers. When Layer 1 criteria are satisfied, family input is documented for communication and conflict mediation but is not decision. Mandatory ethics consultation is triggered when verifiability, applicability, revocation status, or textual clarity is contested (e.g., ambiguity, authenticity disputes, or high-risk disagreement that raises legal/ethical exposure). Even when an AD is legally recognized, bedside enforceability still requires rapid verification of validity and applicability (e.g., scope of the directive, later-in-time updates, and whether the current intervention falls within the documented refusal/consent). Clinicians therefore often engage families briefly as a practical step to confirm relevant facts and to de-escalate conflict and downstream complaints. In the APDM, family input is treated as an information-and-process step—not a veto: persistent objections trigger ethics consultation only when they raise plausible validity/applicability concerns or high legal/ethical exposure. Arguments based solely on third-party interests (e.g., “the patient is needed by the family/society”) are treated as a communication/mediation issue rather than a validity/applicability ground and therefore do not change decisional authority under Layer 1. Any departure from the default autonomy-prioritized pathway must follow the documented trigger-and-escalation steps above; informal overrides based on convenience or pressure are treated as non-compliant and therefore outside the workflow’s protections.

### Layer 2 — appointed proxy/guardian (substituted judgment)

Entry criteria. Layer 2 activates when no controlling directive is available under Layer 1 and a valid appointed proxy/guardian exists.

Justification. Proxy authority is representational and fiduciary in nature: it operationalizes substituted judgment based on the patient’s known values [30] and prior expressions rather than the proxy’s personal preference. Legally, we place an appointed proxy/guardian above default kin because the appointment is an explicit act of patient delegation (agency/voluntary-guardianship logic). In frameworks that formalize delegated healthcare decision-making, a valid appointed agent is typically treated as the primary surrogate, while family-based surrogacy functions as a fallback mechanism when no such appointment exists (e.g., the LPA model under the Mental Capacity Act 2005 in England & Wales; the healthcare agent model under Taiwan’s Patient Right to Autonomy Act).

Ethically, this ordering better protects relational autonomy: the patient’s choice of a trusted representative provides stronger evidence of will than kin status alone and reduces conflicts of interest that can arise in purely default-kin decision-making.

Mandatory ethics triggers. Ethics consultation is mandatory when there is credible divergence from the patient’s known values, a proxy–family conflict, uncertainty regarding the proxy’s authority, or other indicators of heightened risk (e.g., suspected conflicts of interest or contested factual premises). In these situations, ethics escalation functions as a procedural safeguard to prevent value-misaligned substituted judgment and to protect clinicians from unilateral determinations in high-dispute cases.

### Layer 3 — default kin (gap-filling surrogate authority)

Entry criteria. Layer 3 applies only when neither a controlling directive (Layer 1) nor a valid appointed proxy (Layer 2) is available.

Justification. Default kin-based surrogacy is treated as a gap-filling mechanism when the patient’s own verifiable preferences are unavailable. In Korea, the statutory family hierarchy applies and typically requires intra-tier consensus. In China, default authority follows guardianship principles and/or applicable local policy.

What counts as “disagreement” and ethics triggers. “Disagreement” includes (a) conflicts among same-tier relatives, (b) disputes over who holds surrogate authority, and/or (c) conflicts between the surrogate(s) and the clinical team regarding relevant facts, prognosis, or appropriateness. Any of these disagreements triggers mandatory ethics consultation.

### + 1 Execution layer — order translation and auditability

Once a decision source is identified through Layers 1–3 (or via emergency override), the + 1 layer translates the outcome into executable medical orders (e.g., POLST/DNR) and completes EHR-gated documentation. Order entry is conditioned on completion of mandatory fields and an auditable record of verification steps, decision authority, timestamps, and responsible clinician sign-off. Three conflict-resolution rules govern translation: (i) latest-in-time precedence when multiple directives exist; (ii) a clear directive prevails over proxy interpretation; and (iii) emergency override permits stabilization-first action with completion of Duty-to-Search (DtS) and documentation post-stabilization.

### Jurisdictional settings (KR / CN-Local / CN-No-Rule)

Jurisdictional mode modifies how layers are operationalized. KR mode mandates registry queries and use of statutory forms; CN-Local recognizes record-annotated directives, with family objection documented but non-decision when Layer 1 criteria are met; CN-No-Rule does not presume nationwide enforceability and therefore treats ADs as primary evidence, requiring mandatory ethics consultation before withdrawal decisions in high-risk conflicts.

In CN-No-Rule, directives are never treated as automatically controlling; they function as value evidence, and any withdrawal/limitation decision that relies on directive evidence under conflict conditions is routed through mandatory ethics consultation.

### Duty-to-search protocol

Table 2 operationalizes the ethical obligation to seek patient wishes through time-bound, auditable actions. The protocol addresses “passive non-compliance”—clinicians defaulting to family consent without searching for directives.

**Table 2:** Duty-to-Search Protocol: Auditable Compliance Metrics

Action	Timeframe	Responsible	Documentation	Compliance
AD Search	ED: 30–60 min post-stabilization < br> ICU/Ward: 2 h post-admission	Attending/Chief Resident	Search completed (Y/N)	Timestamp required
Registry Query	During AD search	Nurse/Unit Clerk	Query completed (Y/N)	If ‘N’, justify
Family Inquiry	During AD search	Physician/Nurse	Inquiry completed (Y/N)	If ‘N’, justify
Document Verification	Upon any AD discovery	Primary Nurse/Physician	AD status and type	Update from ‘Unknown’
Ethics Consult	Upon conflict/ambiguity	Any team member	Consult triggered (Y/N)	Auto-trigger if conflict

Complete EHR field specifications and documentation templates available in Supplementary Table S2. The ED (30–60 min post-stabilization) and ICU/Ward (within 2 h of admission) windows are configurable default targets used for auditable compliance and quality review. When the patient is conscious and has decision-making capacity, DtS includes direct patient confirmation (whether the AD remains consistent with the patient’s current wishes); any change is documented as a revocation/update prior to order execution

The protocol establishes strict timeframes (ED: 30–60 min post-stabilization; ICU/Ward: 2 h post-admission), assigns clear responsibilities (Attending as initiator, nurses/clerks as executors), and mandates EHR field completion. Inaction leaves a verifiable digital footprint through “Unknown” status defaults. Quality assurance involves monthly field-completeness audits, quarterly time-window compliance checks, and annual ethics consultation reviews.

DtS exceptions and required documentation. The DtS protocol is designed to remain auditable under common failure modes. Registry outages or timeouts are recorded as Registry_Query_Completed = “N” with a brief justification (e.g., “system down/timeout”) and timestamp, while the remaining DtS steps proceed in parallel (EHR search and Family/Proxy Inquiry). Missing or incomplete records are handled by leaving AD_Status = “Unknown” until verification is possible; where conflict flags are present, the workflow routes to ethics consultation via the existing trigger logic rather than allowing premature order translation. Missed time windows do not terminate the protocol: completion is still required with timestamps, and late completion is surfaced through the quarterly time-window compliance review. After emergency override, DtS completion is required post-stabilization: the team completes and timestamps the pending search steps, updates AD_Status from “Unknown” when verification becomes available, and documents any resulting conflict resolution via the ethics trigger pathway.

One illustrative application demonstrates the protocol’s operability. An 80-year-old COPD patient arrives at ED requiring intubation. Family refuses, stating “he wouldn’t want this.” The following timeline (Table 3) shows how the DtS protocol resolves this conflict:

**Table 3:** DtS Protocol Application Timeline (ED Acute Respiratory Failure)

Time	Action	Key Finding
T + 0	Patient arrival, stabilization begins	Family at bedside
T+20 m	DtS protocol initiated; family inquiry completed	Family refuses intubation
T+25 m	EHR search performed	Written AD found in cardiology records
T+30 m	AD verified: “I refuse mechanical ventilation under any circumstances”	Document signed/witnessed
T+45 m	DNI order issued per Layer 1 authority	Process completed within window

Full timeline with responsible persons and EHR fields in Supplementary Table S3

The framework successfully navigated this high-conflict situation, demonstrating how, in local-enabled CN settings (e.g., Shenzhen-style medical-record annotation regimes), DtS-verified documentation can support honoring patient wishes despite family objection, with complete documentation captured within the 30-minute ED window.

### Safe-harbor provisions

Even where ADs are formally recognized, clinicians may still face substantial bedside risk because enforceability depends on documentation checks and procedural safeguards, and dissatisfied relatives may initiate complaints or legal challenges even when the clinician ultimately prevails. Accordingly, the framework addresses clinicians’ “dual fear”: litigation risk for honoring an AD (perceived as “accelerating death”) versus liability risk for disregarding it (e.g., unwanted treatment or battery). We use “safe harbor” in a modular, compliance-based sense: these provisions create rebuttable presumptions (or evidence-weighting effects) when the Duty-to-Search (DtS) protocol and the Autonomy-Prioritized Decision Model (APDM) are followed in good faith with auditable documentation. Importantly, safe-harbor language is not a legal precondition for the framework to operate. Hospitals can implement the workflow as a standard operating procedure under existing law; the safe-harbor templates are offered as implementation aids that strengthen documentation, internal accountability, and (where available) civil-liability clarity.

### Two templates maximize adoptability without assuming legislative change

(1) Institutional Bylaw Model (immediately deployable). This template can be adopted at the hospital/health-system level without statutory amendment. Its primary function is to (i) reduce exposure to institutional sanctions when clinicians act in documented good faith, and (ii) generate litigation-ready compliance records (verification steps, escalation triggers, timestamps, responsible sign-offs) that support a procedural defense. For avoidance of doubt, the Institutional Bylaw Model provides internal protection (e.g., reduced exposure to institutional sanctions) and documentation-based evidentiary defense; it does not confer civil or criminal immunity.

(2) Legislative Model (optional local policy proposal). Where local legislatures or regulators are willing to clarify standards, a legislative template can codify a compliance-based rebuttable presumption in civil contexts, while expressly excluding gross negligence and criminal conduct. This model is presented as a policy option—not a prerequisite—and is intended to reduce uncertainty by linking procedural compliance to evidentiary protection.

Both templates draw on the general principle—reflected in international practice—that procedural compliance and documentation can function as a bridge between ethical justification and legal defensibility (e.g., good-faith action within a recognized decision-making pathway). Consistent with the staged adoption pathway discussed later (Sect.  5.3), institutions can begin with SOP/bylaws (Stage 1), while legislative clarification—if pursued—serves as a later enhancement rather than a condition of feasibility.

Limits and anti-abuse safeguards. These clauses are not “immunity” provisions and do not authorize deviation from governing law. Protection is contingent on (i) a valid decision basis (a verifiable directive where controlling, or documented surrogate consensus where applicable), (ii) DtS completion or justified time-limited emergency override with post-stabilization completion, (iii) auditability requirements that prevent shortcuts, and (iv) mandatory ethics consultation when verifiability, applicability, authority, or interpretation is disputed. Complete model clauses for both templates are provided in Supplementary Text S1. (v) additional high-risk safeguards for treatment limitation/withdrawal decisions under residual ambiguity: a second-clinician confirmation and/or a time-limited therapeutic trial with explicit withdrawal criteria (typically ethics-mediated) to prevent checklist-driven premature withdrawal.

### Framework walkthrough evaluation (Internal Consistency Testing)

#### Integration overview

The four tools create a closed-loop system where legal priority, evidence-gathering, liability protection, and clinical operationalization reinforce each other. The jurisdictional toggle mechanism ensures seamless adaptation across Korea’s statutory regime, China’s local rules, and non-rule jurisdictions while maintaining internal consistency.

#### Scenario testing results

We evaluated internal coherence through a scenario-based walkthrough of 12 scenarios spanning three clinical settings, three jurisdictions, and five conflict types (Table 4).

**Table 4:** Scenario Walkthrough Summary (abbreviated)

ID	Setting	Jurisdiction	Conflict Type	Resolution Pathway	HEC Required
S1	ED	KR	No-AD	Layer 3 → HEC	Yes
S2	ED	CN-Local	Emergency-Incomplete	Emergency Override → Layer 1	No
S3	ED	CN-No-Rule	Ambiguous AD	Layer 3 → HEC	Yes
S4	ICU	KR	AD Exists	Layer 1 (AD enforced)	Yes
S5	ICU	KR	Duplicate AD	Layer 1 (Latest-in-Time)	No
S6	ICU	CN-Local	Proxy-vs-Kin	Layer 2 (Proxy prevails)	Yes
S7	ICU	CN-No-Rule	No-AD	Layer 3 → HEC	Yes
S8	Ward	KR	Ambiguous AD	Layer 1 fail → Layer 3	Yes
S9	Ward	CN-Local	AD Exists	Layer 1 (AD enforced)	Yes
S10	Ward	CN-No-Rule	Proxy-vs-Kin	Layer 3 → HEC	Yes
S11	ED	KR	Emergency-Incomplete	Emergency Override → Layer 1	No
S12	Ward	CN-No-Rule	Ambiguous AD	Layer 3 → HEC	Yes

HEC=Hospital Ethics Committee (used here as an umbrella term for hospital-based ethics consultation, which may be delivered by an ethics committee, ethics consultation service, or MDT, depending on institutional capacity). Detailed scenario descriptions, conflict narratives, and resolution outcomes available in Supplementary Table S4. Scenario construction and literature-informed sampling are described in Sect. 3.3.

Summary Statistics: We conducted two walkthrough rounds: Round 1 applied the v1 framework; documented divergences (primarily applicability parsing ambiguities) prompted refinement, after which Round 2 reran all 12 scenarios and achieved full convergence. Path convergence: 83% (first round) → 100% (after refinement). HEC consultation triggered: 75% (9/12). All scenarios achieved mode-consistent, legally traceable resolutions under the active toggle (with CN-No-Rule treating directives as value evidence and routing high-risk withdrawal/limitation decisions through mandatory ethics consultation).

Illustrative Example S4 (KR, ICU; verifiable AD and family objection): A 75-year-old man with metastatic cancer is admitted to the ICU and lacks decision-making capacity. Under the Duty-to-Search (DtS) protocol, the clinical team queries the national registry and identifies a valid Korean statutory AD (Form No. 6) in which the patient refused chemotherapy. The patient’s adult children, unaware that an AD exists, object to limitation of treatment and demand “the best available treatment,” raising the possibility of complaint or litigation. Under the KR jurisdictional mode, the APDM enters Layer 1 (Verifiable AD) because the directive is registry-verified and directly applicable to the contested intervention. Family objection is therefore documented but is not decision once the Layer 1 criteria are met. Given the intensity of conflict and the need for structured communication, the case triggers HEC consultation to mediate the dispute and clarify the clinicians’ legal obligations under the LSTDA framework. Following the ethics-facilitated family meeting, the care plan is translated into executable orders, including issuance of the relevant POLST (Form No. 13) reflecting comfort-focused care consistent with the verified directive. The team records DtS completion (registry query), the directive source, the ethics conclusion, and responsible sign-offs in the EHR, enabling the safe-harbor documentation pathway to support the physician’s good-faith compliance.

Illustrative Example S6 (CN-Local, ICU; Proxy-vs-Kin over PEG): A 68-year-old woman is admitted to the ICU after a severe stroke and loses decision-making capacity. Under the Duty-to-Search (DtS) protocol, the clinical team confirms that no controlling, record-annotated advance directive resolves the immediate question. The patient’s daughter presents documentation establishing that she is the patient’s appointed proxy and refuses placement of a percutaneous endoscopic gastrostomy (PEG) tube, stating that the patient had consistently rejected long-term artificial feeding. The patient’s spouse, who would otherwise act as the default kin surrogate, disagrees and demands PEG placement. Because no controlling directive governs this decision, the case enters Layer 2 (Appointed Proxy); however, the proxy–kin conflict triggers mandatory ethics consultation. The ethics committee reviews the proxy documentation and confirms the daughter’s authority under applicable local rules, and evaluates whether the proxy decision reflects substituted judgment rather than personal preference. After the ethics consultation, the team documents DtS completion, the basis for proxy authority, the spouse’s objection, and the ethics conclusion in the medical record. Consistent with Layer 2, the proxy decision is upheld, and the care plan is translated into executable orders with audit-ready documentation.

Illustrative Example S12 (CN-No-Rule, Ward; ambiguous directive and intra-family conflict): A 90-year-old man with advanced dementia is admitted to the ward and lacks decision-making capacity. The Duty-to-Search (DtS) protocol identifies a handwritten note stating, “don’t let me suffer,” but the document is ambiguous and does not specify the treatments to be limited or the clinical conditions under which limitations should apply. Family members disagree sharply: the patient’s son argues that the note means all life-prolonging treatment should be stopped immediately, while the daughter insists on full care. In the CN-No-Rule mode, the directive is treated as strong evidence of the patient’s values rather than a controlling order, and because there is no valid appointed proxy to resolve the dispute, the case proceeds to Layer 3 (Default Kin). Given the intra-family disagreement and the high-stakes nature of the decision, the model requires mandatory HEC consultation. The ethics committee reviews the note as value evidence, facilitates a structured discussion, and mediates a compromise: a time-limited trial of treatment with explicit, pre-specified withdrawal criteria tied to the patient’s condition and goals of care. The agreed plan is then documented and translated into executable medical orders, with DtS completion, conflict flags, and the ethics conclusion recorded to create an auditable rationale under the CN-No-Rule pathway.

Additional scenarios (Supplementary Table S4) demonstrated similar patterns: CN-Local proxy appointments prevailing over default kin preferences, and CN-No-Rule ambiguous directives triggering mandatory ethics mediation with time-limited therapeutic trials.

#### Key findings

Consistent with the iterative validation process described in Sect.  3.3, path convergence improved from 83% (10/12) to 100% after iterative refinement addressing applicability parsing ambiguities. This 100% convergence reflects internal consistency after iterative refinement of the decision logic, not external validation of real-world usability or clinical effectiveness. The added “Applicability Checklist” resolved whether “mechanical ventilation” includes post-operative support and whether “no prolonged suffering” encompasses PEG placement. The full Applicability Checklist (including the three mandatory questions and worked examples used in the walkthrough refinement) is provided in Supplementary Table S5. To support implementation, we also publish a consolidated if–then rule set (Supplementary Table S6) that operationalizes DtS execution, toggle selection (KR/CN-Local/CN-No-Rule), APDM layer selection, and + 1 order translation as implementation-ready specifications for the core tools.

Ethics consultations triggered appropriately in 75% (9/12) of scenarios. Three exceptions were emergency overrides with post-stabilization resolution (S2, S11) and unambiguous latest-in-time applications (S5). All scenarios achieved mode-consistent, legally traceable resolutions—not emotional satisfaction but clear decisions grounded in the active toggle’s recognition pathway with documented justification; for CN-No-Rule, “traceability” denotes consistency with principles-level consent/guardianship norms plus mandatory ethics escalation, without implying nationwide Chinese AD enforceability or guaranteeing litigation outcomes.

The jurisdictional toggle mechanism demonstrated robustness: Korean scenarios utilized statutory forms and registry; CN-Local scenarios applied medical record annotation rules where local enabling regimes exist; CN-No-Rule scenarios mandated deliberative ethics processes. Across all modes, the walkthrough identified no mode-inconsistent steps under the stated assumptions, supporting the framework’s adaptive capability.

### Summary

The framework provides an operational solution to AD-family conflicts through jurisdiction-aware decision architecture, process-auditable evidence gathering, and aligned liability protection. By converting ethical aspirations into verifiable bedside actions, it clarifies who decides, ensures evidence is sought and recorded, and specifies resulting orders with documented legal and ethical basis. The scenario testing confirms theoretical operability across diverse contexts, providing a validated design ready for empirical implementation. Future mixed-methods pilots will evaluate real-world performance, clinician acceptance, and patient outcomes under appropriate ethics review.

## Discussion

Our normative framework provides an operational, legally-grounded solution to AD-family conflicts in China and Korea. By translating abstract principles into concrete bedside procedures, it bridges the gap between legal theory and clinical practice while respecting both individual autonomy and relational values.

### Principal contributions

This study makes three key contributions to the field. First, the Autonomy-Prioritized Decision Model moves beyond simplistic “AD-first” approaches by providing structured pathways for real-world scenarios where directives are missing, invalid, or ambiguous. Rather than excluding families, it integrates their participation within a mode-consistent hierarchy. Second, the jurisdictional toggle mechanism addresses East Asia’s legal pluralism, enabling seamless adaptation across Korea’s codified system, China’s local-enabled jurisdictions, and principles-only environments. This flexibility reflects the gradualist nature of legal reform in the region. Third, the auditable DtS protocol converts soft ethical obligations into hard procedural requirements with mandatory EHR fields and timestamps, creating both quality improvement datasets and legal defense documentation.

### Implementation pathways

Our implementation pathways align with established implementation science frameworks. The Consolidated Framework for Implementation Research (CFIR) [31]identifies key determinants we address: intervention complexity (jurisdiction settings), outer setting (legal environment), and process (staged adoption) [32].

### China: gradualist adoption

Without national legislation, China requires a bottom-up approach [33]. Stage 1 enables immediate hospital-level adoption as standard operating procedures with institutional bylaws providing internal protection. Stage 2 leverages local legislative powers in pioneering jurisdictions like Shenzhen to create statutory safe-harbors. Stage 3 uses accumulated evidence to support eventual national codification. This pathway acknowledges political realities while enabling incremental progress.

### Korea: optimizing existing law

Korea’s comprehensive LSTDA requires enhanced implementation rather than new legislation [34]. Empirical studies have documented specific implementation challenges: decision-making processes for cancer patients have fundamentally changed yet remain problematic [35, 36], enforcement varies significantly across clinical contexts [37], persistent gaps exist between survey data and clinical realities [38], and unintended consequences like reduced organ donation rates have emerged [39]. Critical analyses have synthesized these operational problems [40] and proposed systematic improvements based on multi-year implementation experience [41]. Our framework addresses these documented gaps by adding substantive compliance checks beyond formalistic verification, clarifying mandatory ethics consultation triggers, and enabling institutional-level quality auditing.

### Addressing implementation barriers and risks

Four predictable barriers require mitigation strategies. Implementation research has documented similar barriers in naïve contexts [42], with the COVID-19 pandemic exacerbating existing challenges [43]. Healthcare professional surveys in Singapore reveal system-level barriers that resonate with our framework’s challenges [44]. Technical integration challenges can be addressed through phased approaches: paper-based minimum viable products for legacy systems, standardized data exchange protocols, and eventual cloud-based registries. Human capacity limitations necessitate targeted HEC training programs, with our clear Layer 1–2 rules resolving most cases without ethics consultation.

Even when “ethics consultation” is required, we do not assume that a standing hospital ethics committee (HEC) will have the time or staffing to conduct prolonged family mediation in every high-conflict case. In practice, ethics capacity is uneven, and family-facing work often falls on a small number of trained individuals rather than the full committee. To make the escalation pathway realistic, “mandatory ethics consultation” should be understood as mandatory ethics support, which can be delivered through a multidisciplinary ethics response team (MDT) with escalation to a formal HEC determination when needed. Consistent with Krishna’s work emphasizing multidisciplinary structures for end-of-life conflict management, an MDT can combine complementary skill sets for both value-clarification and family communication [45] (e.g., attending physician/ICU lead, bedside nurse or nurse manager, palliative care clinician, social worker, a clinical ethicist/HEC member, and—where available—legal/risk liaison and chaplaincy/spiritual care). Triage can be performed by an on-call ethics lead or designated “ethics liaison” within a short time window to determine whether the case is (i) resolvable via structured family meeting and documentation by the MDT, or (ii) requires escalation to the full HEC. Escalation should be reserved for high-stakes, high-dispute triggers such as contested decision authority (proxy validity/kin hierarchy), ambiguous or disputed directive applicability/authenticity, suspected conflicts of interest or coercion, threatened litigation/administrative complaints, or disagreements over withdrawal criteria after a time-limited trial.

Cultural resistance is addressed primarily through safe-harbor protections that reduce clinician exposure to social backlash while documented compliance provides evidence of good faith. Economic misalignment requires policy advocacy for reimbursable advance care planning and quality metrics tied to AD execution rather than ICU occupancy.

Larger ethical and existential stakes. End-of-life decisions inevitably implicate deeper philosophical and spiritual questions about the meaning of life and death, the moral weight of suffering, and what it means to respect a person when they can no longer speak for themselves. Reasonable people (and traditions) disagree on whether the primary ideal is the sanctity-preservation of life, the avoidance of non-beneficial suffering, or dignity understood as fidelity to a person’s values and narrative identity. Rather than resolving these ultimate disagreements at the bedside, law and institutional systems should provide procedural justice: a transparent, reviewable process that (i) preserves the patient’s verifiable voice when available, (ii) disciplines surrogate influence into accountable roles, and (iii) protects clinicians from ad hoc bargaining under moral duress. Our workflow is designed to operationalize this procedural ideal in pluralistic settings, allowing different value commitments to be expressed through the patient’s documented choices while maintaining fair, auditable safeguards.

Three ethical risks merit explicit attention. Cost-driven premature withdrawal is prevented through bidirectional search requirements (for both ADs and revocation evidence), exclusion of bad-faith decisions from safe-harbor protection, and mandatory ethics review in CN-No-Rule jurisdictions. Family conflict exacerbation is mitigated through required communication touchpoints, HEC mediation focused on relational repair, and bereavement support referrals. Vulnerable patient coercion is addressed through voluntariness verification, automatic ethics review for suspicious directives, and strict applicability checks preventing misuse of generic statements.

Prolonging life versus burdens of treatment. A recurring dilemma is whether medicine should act to extend life when doing so requires substantial short-term suffering. In this framework, the answer depends on capacity and applicability rather than a blanket rule. When the patient has decision-making capacity, contemporaneous informed preferences govern (including reconsideration of prior AD statements). When the patient lacks capacity and a directive is verifiable and applicable, it governs, reflecting the ethical stance that autonomy can include refusing burdensome life-prolongation. When applicability is uncertain or when a reversible intervention is needed to create time for verification and deliberation, the workflow permits time-limited stabilization (Emergency Override) and, in contested cases, time-limited trials with explicit withdrawal criteria through ethics consultation—ensuring that any temporary deviation remains bounded, documented, and aligned with the patient’s values once clarified.

Ethical comparison with plausible alternative workflows. Several alternative flows were plausible at the design stage. A first alternative is an autonomy-absolutist flow (“a clear AD always governs; no family-facing step”), which maximizes formal autonomy but often fails under bedside enforceability constraints (verification/applicability uncertainty, conflict escalation, and institutional risk), potentially producing covert non-compliance rather than principled adherence. A second alternative is a family-dominant flow (“family preferences may override an AD”), which may align with some relational practices but systematically weakens the patient’s verifiable voice and risks overtreatment that defeats advance planning. A third alternative is a committee-first flow (routing most conflicts directly to ethics committees), which may enhance deliberation but is slow and resource-intensive, and is not scalable in time-pressured ED/ICU contexts.

By contrast, our proposed workflow is autonomy-prioritized but not autonomy-exclusive: it preserves the AD as the governing authority once verifiable and applicable, while structuring family involvement as an information-and-process step (not a veto) and triggering ethics escalation only under predefined criteria. It also encompasses scenarios that the above alternatives handle poorly, including jurisdictional heterogeneity (KR/CN-Local/CN-No-Rule), ambiguous or generic directives requiring applicability parsing, contested surrogate authority, and urgent instability managed through time-limited stabilization gates followed by documented alignment. These alternatives and tradeoffs are reflected in the safeguards and escalation logic described in Sect.  4.2–4.4.

### International comparison and theoretical contribution

Building on the cross-jurisdictional analysis (Sects.  2–3) and the 12-scenario walkthrough results (Sect.  4.5), this study makes three theoretical contributions. First, we advance the concept of operational autonomy—translating autonomy commitments into auditable bedside actions and documentation checkpoints. In our framework, this is instantiated through the DtS protocol (time-bounded search and verification steps), the APDM layer logic (who governs, when, and how), and the executable “+1” translation that converts verified directives into chart-ready orders. Second, we introduce jurisdictional plasticity as a design principle for legal–clinical interfaces in heterogeneous regulatory environments. This is operationalized through the jurisdictional toggle (KR / CN-Local / CN-No-Rule), allowing the same clinical workflow to remain legally traceable while adapting verification and escalation requirements to local enforceability conditions. Third, we demonstrate normative design methodology as a systematic approach for converting legal–ethical principles into auditable workflows; importantly, our two-round scenario walkthrough (Round 1 → documented divergences → refinement → Round 2 convergence) illustrates how normative rules can be iteratively specified and stress-tested for internal coherence before future usability and implementation studies.

Internationally, our design draws on selected functional anchors rather than attempting an exhaustive country-by-country comparison. From the UK Mental Capacity Act (2005), we adapt the “reasonable steps” logic into an auditable DtS protocol and retain a structured route for handling uncertainty and disagreement. From Taiwan’s Patient Right to Autonomy Act (2019), we draw on the operational logic of advance care planning and registry-linked discoverability, while offering a workflow that is intended to be scalable even where resource-intensive ACP consultations are not uniformly available. We also situate these anchors against the two primary jurisdictions analyzed in this paper: South Korea’s codified statutory pathway (forms and national registry) and China’s variable landscape (local enabling rules or none), which together motivate the need for a jurisdiction-adaptive, audit-ready bedside workflow.

Theoretically, the framework contributes to culturally responsive bioethics by showing that autonomy can be implemented without excluding relational values. Family involvement is structured as an information-and-process step within a clear hierarchy rather than as an ad hoc bargaining mechanism. Methodologically, it contributes to the emerging intersection of healthcare law, ethics, and informatics by providing a replicable pathway from principles to auditable actions. Practically, it offers a jurisdiction-adaptive protocol that can be evaluated in future clinician-facing studies for usability, time cost, and implementation effectiveness.

### Limitations and future research

This normative design study establishes theoretical operability as the necessary foundation for evaluating empirical effectiveness. Our systematically constructed scenarios provide structured coverage of key decision pathways; real-world variations will enrich rather than invalidate the framework. While focused on China and Korea, intra-cultural variation and applicability to other East Asian contexts remain untested.

A four-phase research agenda should follow. Phase 1 involves qualitative feasibility assessment through focus groups with clinicians and ethics committees. Phase 2 implements pilot studies measuring compliance rates, decision times, and stakeholder satisfaction. Phase 3 conducts multi-center trials comparing framework hospitals to usual care on AD execution fidelity, family satisfaction, and clinician moral distress. Phase 4 analyzes policy impact through interrupted time-series studies after legislative adoption.

###  Discussion Summary

End-of-life decision-making will always involve profound complexity at the intersection of autonomy, relationships, law, and culture. This framework does not claim to make difficult decisions easy, but rather to make them clear, just, and accountable. By providing structured pathways for honoring patient wishes while engaging families, auditable procedures for evidence-gathering, and protection for good-faith compliance, it offers a substantial improvement over the current ad-hoc, fear-driven status quo.

The framework’s ultimate value lies not in resolving all conflicts but in creating space for essential dialogues: between patients and families about final wishes, between clinicians and families grounded in respect for patient voice, and between society and the reality of death. Through operational clarity and procedural justice, we can better honor the most personal choices a human being makes while respecting the relational bonds that give those choices meaning.

## Conclusions

End-of-life (EOL) medical decision-making represents one of the most profound moral crucibles of modern society. When a patient’s advance directive (AD) clashes with the wishes of their family, clinicians are trapped in a multi-dimensional dilemma of law, ethics, and culture. This study has addressed this core challenge by proposing a normative design framework, engineered to provide an operational, legally-grounded, and ethically-defensible solution for healthcare institutions in China and Korea.

Our core contributions are threefold. On a theoretical level, this research moves beyond the simplistic binary of individual versus relational autonomy. Through a functional comparative analysis, we first identified the fundamental divergence in the legal foundations of EOL decision-making—Korea’s “codified” pathway versus China’s “principles-based + local-breakthrough” model. We then proposed a concept of “layered autonomy,” which reconciles these tensions by structuring, rather than excluding, relational values within a clear hierarchy.

On a design level, we translated these abstract principles into concrete tools. We created the Autonomy-Prioritized Decision Model, which converts legal ambiguity into an unequivocal decision hierarchy. We developed the auditable “Duty-to-Search” (DtS) protocol, transforming the vague ethical expectation to search for an AD into a measurable procedural standard. Finally, we designed robust “Safe-Harbor” clauses that shield clinicians who follow this procedure from the threat of undue litigation.

On a practical level, this framework provides implementation-ready tools, including policy texts, workflow charts, and EHR field templates. Critically, we designed “jurisdiction settings” that allow the framework to remain robust and legally compliant across all three identified legal environments (KR, CN-Local, CN-No-Rule). The conceptual validation via scenario-based walkthroughs confirmed the framework’s logical coherence and operational feasibility.

We must, however, be explicit about this study’s limitations. This is a normative design study, not an empirical validation. We have demonstrated that the framework is theoretically coherent, legally compliant, and conceptually operable. We cannot claim, at this stage, that it will be adopted by clinicians, that it will effectively reduce bedside conflict, or that it will improve the qualitative experience of patients and families. These critical questions must be answered by future mixed-methods research.

Ethical humility and governance. Because end-of-life decisions are morally weighty and often irreversible, this framework should be understood as decision-support rather than decision replacement. It does not resolve ultimate disagreements about the meaning of life, death, or suffering; instead, it aims to provide procedural safeguards—verifiability, accountability, and reviewability—so that plural moral commitments can be expressed through the patient’s documented preferences and handled consistently under uncertainty. Accordingly, implementation should include clinician training, institutional oversight (e.g., ethics consultation capacity), and prospective evaluation of usability, error modes, and unintended consequences, with iterative refinement based on real-world feedback.

This framework provides a rigorously developed, legally grounded blueprint ready for implementation. Its strength lies precisely in its normative clarity—uncompromised by the political negotiations that often dilute bedside protocols. Hospitals can adopt it immediately as policy, legislators can reference it for reform, and researchers can use it as a well-specified design for empirical testing. By establishing clear precedence rules, auditable procedures, and protective mechanisms, we provide the operational infrastructure necessary for honoring patient autonomy within relational contexts.

## Supplementary Information

Supplementary Material 1.

## Data Availability

All data generated or analysed during this study are included in this published article and its supplementary information files.

## Abbreviations

AD: Advance directive
POLST: Physician Orders for Life-Sustaining Treatment
LSTDA: Act on Decisions on Life-Sustaining Treatment for Patients at the End-of-Life
HEC: Hospital Ethics Committee
EHR: Electronic health record
KPI: Key performance indicator
