# Supplementary material for: When advance directives clash with family consent: designing an operational framework for end-of-life decision-making in China and Korea
Source: BMC Med Ethics. 2026 Apr 1;27:92. doi: 10.1186/s12910-026-01451-1 (PMC13169564; doi:10.1186/s12910-026-01451-1)
Supplement: Supplementary file 1 — Supplementary Material 1. [file 12910_2026_1451_MOESM1_ESM.docx]

**Supplementary Table S1：**

**Table 1. Core Operational Elements of End-of-Life Decision-Making Frameworks**

| **Legal Element** | **China (Civil Code & Local Regulations)** | **South Korea (LSTDA & Implementing Rules)** | **Design Implication** |
| --- | --- | --- | --- |
| **Legal Status of AD** | No unified national statute; principles-level framework; enforceability relies on local regulations. [a] | Formally recognized and legally binding if compliant with statutory form. [b] | Center AD primacy where enabled; otherwise treat as strongest evidence + Ethics pathway. |
| **Formal Requirements** | National: general principles; Local: written/audiovisual, clear expression, medical record annotation. [c] | Statutory forms; consultation and registration required. [d] | Prioritize verification of authenticity and capacity at signing; add checkpoints. |
| **Default Surrogate Order** | No specific national hierarchy; relies on guardianship principles; local/hospital policy may specify. | Explicit hierarchy: Spouse → Lineal descendants → Lineal ascendants → Siblings; intra-tier consensus required. [e] | Predictability is needed but remains subordinate to AD/proxy. |
| **Dispute Resolution** | No national dedicated mechanism; civil litigation or local ethics committees if mandated. | Institutional HECs mandated; courts as last resort. [f] | Rapid, hospital-based mechanism is essential. |
| **Safe Harbor for Clinicians** | No explicit national provision; contingent on adoption in local law/bylaws. | Compliance with LSTDA procedures affords strong defense (not labeled “safe harbor”). | Tie liability protection to auditable procedural compliance and formal adoption. |

Notes:

[a] PRC Civil Code (Personality Rights; Book VII—Tort Liability, incl. Art. 1219). Enforceability depends on local rules; Shenzhen Special Economic Zone Medical Regulations (2022 rev.) Art. 78.

[b] LSTDA, Article 10 (Preparation of Advance Directives on Life‑Sustaining Treatment).

[c] Local rules often require written or audiovisual form, clear expression, and medical record annotation.

[d] Enforcement Rule standard forms: Form No. 6 (AD) and Form No. 13 (POLST), administered by the National Agency for Life‑Sustaining Treatment Decisions.

[e] LSTDA, Article 17 (Surrogate hierarchy and intra-tier consensus).

[f] LSTDA, Article 14 (Institutional Ethics Committees).

**Supplementary Table S2:**

**Table 2. Duty-to-Search Protocol: Auditable Compliance Metrics:**

| **Metric** | **Timeframe** | **Responsible Person** | **EHR Documentation Field (Example)** | **Compliance Determination** |
| --- | --- | --- | --- | --- |
| **Initial Search** | ED: within 30–60 minutes post-stabilization | Attending Physician / Chief Resident (Initiator) | Initial_Search_Timestamp | Documented completion within timeframe. |
| **Comprehensive Search** | ICU/Ward: within 2 hours of admission | Attending Physician / Chief Resident (Initiator) | Comprehensive_Search_Timestamp | Documented completion within timeframe. |
| **Registry Query** | Part of Initial/Comprehensive search | Nurse / Unit Clerk (Executor) | Registry_Query_Completed (Y/N; Timestamp) | Binary completion; 'N' requires justification. |
| **Family/Proxy Inquiry** | Part of Initial/Comprehensive search | Physician / Nurse (Executor) | Family_Inquiry_Completed (Y/N; Persons_Asked: ___) | Binary completion; 'N' requires justification. |
| **Documentation Verification** | Upon discovery of any document | Primary Nurse / Physician | AD_Status (Found/NotFound/Unknown); AD_Type (Written/AudioVideo/Registry) | Status updated from 'Unknown'. |
| **Ethics Consultation Trigger** | Immediately upon conflict/ambiguity | Any Team Member (Initiator) | Ethics_Consult_Triggered (Y/N; Ticket_No) | Auto-trigger 'Y' if any key step is incomplete or conflict noted. |

**Supplementary Table S3**

**Table 3 DtS Protocol Application Timeline (ED Acute Respiratory Failure)**

| **Timepoint** | **Action** | **Responsible Person** | **EHR Documentation** |
| --- | --- | --- | --- |
| **T+0** | Patient arrives, stabilization begins. | ED Team | --- |
| **T+15m** | Patient stabilized on high-flow non-invasive ventilation. Intubation decision is imminent. | Attending Physician | --- |
| **T+20m** | Attending initiates DtS protocol. Nurse performs Family Inquiry. | Attending, Nurse | Initial_Search_Timestamp set. Family_Inquiry_Completed=Y. Notes: "Family refuses intubation, states patient's wishes." |
| **T+25m** | Unit Clerk performs EHR / internal hospital record search. | Unit Clerk | AD_Status=Found. AD_Type=Written. Notes: "AD scanned into cardiology record 3 years prior." |
| **T+30m** | Attending verifies AD document. | Attending Physician | AD text clearly states: "I refuse mechanical ventilation or intubation under any circumstances." Document is signed and witnessed. |
| **T+35m** | Attending activates Layer 1 of the "3+1" Architecture. | Attending Physician | Legal_Basis=Layer1_AD. Jurisdiction=CN-Local. |
| **T+40m** | Attending holds brief meeting with family. Explains: "We have found a formal directive from your father. It clearly refuses this treatment. Legally and ethically, we must honor his voice." | Attending Physician | AD_Explained_to_Family=Y. |
| **T+45m** | Attending issues final order. | Attending Physician | POLST_Issued=Y. Order: "DNI (Do Not Intubate). Continue comfort measures and non-invasive support." |

**Result:** The framework successfully navigates a high-conflict, time-sensitive situation. It uses the DtS protocol to find the determinative Layer 1 artifact, which gives the physician the clear legal and ethical authority (under CN-Local rules) to honor the patient's wishes, even in the face of initial family disagreement. The entire process is documented and auditable.

**Supplementary Text S1**

**Complete Safe-Harbor Model Clauses**

The following model clauses provide the full legal text for the "Legislative Model" and "Institutional Bylaw Model" summarized in Section 4.4 of the main text.

**A. Legislative Model (Statutory Provision)** *Draft text intended for adoption by local legislatures or national health ministries.*

**Section X — Safe-Harbor for Good-Faith Compliance in End-of-Life Decisions**

1. A physician, nurse, or other licensed healthcare provider who, in good faith, withholds or withdraws life-sustaining treatment in accordance with an apparently valid Advance Directive, a duly authorized healthcare proxy, or the statutory surrogate hierarchy shall be afforded a rebuttable presumption of good-faith compliance in civil proceedings and professional review, absent gross negligence or willful misconduct.
2. This presumption applies only where such actions are expressly authorized by applicable law and completed pursuant to the Minimum Compliance Checklist, including required ethics consultation when indicated and, where residual ambiguity remains, a second-clinician confirmation and/or a time-limited therapeutic trial with explicit withdrawal criteria.
3. The presumption in Paragraph 1 is rebuttable upon a clear and convincing showing that the provider had actual knowledge of revocation or invalidity, or failed to complete the Checklist.
4. Nothing in this Section precludes administrative or disciplinary review under applicable professional or institutional rules, nor does it affect criminal liability standards under applicable law.

**B. Institutional Bylaw Model (Policy Provision)** *Draft text intended for immediate adoption as internal hospital policy.*

**Policy Y — Procedural Safe‑Harbor for End‑of‑Life Decisions**

1. Providers acting in good faith who complete the hospital’s Duty-to-Search, obtain required Ethics Consult when indicated, and translate decisions into POLST/medical orders shall be presumed compliant with institutional policy (internal protection) and supported by documentation-based evidentiary defense, subject to post-hoc audit; this policy does not create civil or criminal immunity.
2. This presumption is rebuttable upon findings of gross negligence, willful misconduct, or material deviation from the Checklist.
3. Compliance audits will rely on EHR‑captured timestamps and fields listed in the Duty-to-Search Protocol.
4. For treatment limitation/withdrawal decisions under residual ambiguity or high-conflict conditions, the policy requires a second-clinician confirmation and/or an ethics-mediated time-limited trial with explicit withdrawal criteria, documented in the medical record.

**Supplementary Table S4**

**Table 4. Comprehensive Scenario Walkthrough Summary**

| **Scenario ID** | **Clinical Setting** | **Jurisdiction** | **Conflict Type** | **Key Conflict Description** | **Primary Framework Layer Activated** | **Ethics Consult Triggered?** | **Consensus Achieved?** | **Notes** |  |
| --- | --- | --- | --- | --- | --- | --- | --- | --- | --- |
| **S1** | ED | KR | No-AD | 65F cardiac arrest; no registry hit; spouse wants full code, adult son wants DNR | Layer 3 (Default Kin); intra-tier disagreement | **Yes** | **Yes** (after HEC mediation) | HEC facilitated time-limited trial; consensus for DNR after 48h if no recovery |  |
| **S2** | ED | CN-Local | Emergency-Incomplete | 80M COPD respiratory failure; family refuses intubation but AD search incomplete due to time pressure | Emergency Override → Layer 1 (post-stabilization) | No (AD found post-stabilization) | **Yes** | AD discovered at T+25m clearly refusing intubation |  |
| **S3** | ED | CN-No-Rule | Ambiguous AD | 72F stroke; handwritten note "no suffering"; daughter wants comfort care, son wants aggressive treatment | Layer 3 → Mandatory HEC | **Yes** | **Yes** (compromise) | HEC brokered time-limited trial of conservative measures |  |
| **S4** | ICU | KR | AD Exists | 75M metastatic cancer; valid Form No.6 refusing chemo; family objects, unaware of AD | Layer 1 (Verifiable AD) | Yes (family conflict) | **Yes** (AD enforced) | [Detailed in Results 4.5.2] HEC explained legal obligation; Safe-Harbor protected MD |  |
| **S5** | ICU | KR | Duplicate AD | 68F dementia; two ADs found—2020 (wants full treatment) vs 2023 (refuses dialysis); family prefers 2020 version | Layer 1; Latest-in-Time rule applied | No | **Yes** | 2023 AD prevailed after signature/witness verification |  |
| **S6** | ICU | CN-Local | Proxy-vs-Kin | 68F post-stroke; daughter (appointed proxy) refuses PEG; spouse (Layer 3) demands PEG | Layer 2 (Appointed Proxy) prevails | Yes (kin objection) | **Yes** (proxy decision upheld) | HEC confirmed proxy authority under local law |  |
| **S7** | ICU | CN-No-Rule | No-AD | 55M liver failure; no AD; siblings disagree (2 want transplant listing, 1 opposes due to patient's prior "quality over quantity" statements) | Layer 3 → Mandatory HEC | **Yes** | **Yes** (majority + substituted judgment) | HEC weighed prior oral statements; consensus for listing with clear withdrawal criteria |  |
| **S8** | Ward | KR | Ambiguous AD | 82M Parkinson's; Form No.6 states "no prolonged suffering" but unclear if applies to current PEG decision | Layer 1 applicability check failed → Layer 3 | **Yes** | **Yes** (family consensus + HEC guidance) | Applicability checklist revealed conditional language; family agreed to short-term PEG trial |  |
| **S9** | Ward | CN-Local | AD Exists | 90M dementia; clear written AD (annotated in medical record) refusing antibiotics for infections; daughter accepts, son objects citing "changed his mind" (no evidence) | Layer 1 (Verifiable AD) | Yes (son's objection) | **Yes** (AD enforced) | Son could not provide evidence of revocation; DtS protocol documented search for contrary evidence |  |
| **S10** | Ward | CN-No-Rule | Proxy-vs-Kin | 70F cancer; orally designated nephew as proxy (witnessed but not written); nephew wants palliative sedation, siblings want active treatment | Proxy claim not formally recognized as a valid Layer 2 appointment in CN-No-Rule → Layer 3 → Mandatory HEC | **Yes** | **Yes** (HEC-mediated compromise) | HEC treated oral designation as strong evidence but negotiated trial period before sedation |  |
| **S11** | ED | KR | Emergency-Incomplete | 45M trauma; family states "he has an AD somewhere" but registry query times out (system down); needs emergency surgery | Emergency Override → post-op Layer 1 search | No (emergency) | **Yes** (post-op search completed) | Surgery proceeded; registry query completed 6h post-op (AD found, no conflict with surgery decision) |  |
| **S12** | Ward | CN-No-Rule | Ambiguous AD | 90M dementia; handwritten note "don't let me suffer"; son wants all treatment stopped, daughter wants full care | Layer 3 → Mandatory HEC | **Yes** | **Yes** (time-limited trial) | HEC mediated compromise with clear withdrawal criteria |  |

**1. Legend and Definitions**

**Abbreviations: ED, Emergency Department; ICU, Intensive Care Unit; KR, Korea; CN-Local, China with local enabling rules (e.g., Shenzhen); CN-No-Rule, China without specific local AD legislation; HEC, Hospital Ethics Committee; DNR, Do Not Resuscitate; PEG, percutaneous endoscopic gastrostomy.**

**Conflict Types:**

**No-AD: No advance directive found; surrogate decision-makers disagree.**

**AD Exists: Valid AD found but family/kin object.**

**Ambiguous/Duplicate AD: Multiple or unclear directives requiring interpretation.**

**Proxy-vs-Kin: Appointed proxy conflicts with default kin hierarchy.**

**Emergency-Incomplete: Life-threatening situation requiring immediate action before full search completion.**

**2. Extended Walkthrough Findings (Internal Consistency Testing) The following analysis details the iterative refinement process and logical coherence testing conducted during the scenario walkthroughs.**

**Path Convergence: Ten scenarios (83%) produced convergent decision paths between the two independent reviewers on the first walkthrough pass. Two scenarios (S5, S8) revealed initial divergence related to the parsing of "applicability" in ambiguous directive language—specifically, whether a directive declining "mechanical ventilation" encompasses short-term post-operative ventilation (S5), and whether "no prolonged suffering" encompasses PEG tube placement (S8). Importantly, no logical dead-ends or unresolvable decision loops were encountered in any scenario, confirming the framework's internal routing logic.**

**Iterative Refinement: The divergence in S5 and S8 prompted the addition of an explicit "Applicability Checklist" embedded in the Layer 1 workflow, comprising three mandatory questions: (i) Does the directive explicitly mention the present intervention or condition? (ii) Does the directive contain conditional language (e.g., "if terminal," "if no hope of recovery")? (iii) Is the anticipated condition currently present? We also introduced a "Local-Enabled?" guard to prevent misapplication of statutory rules in non-enabling jurisdictions, and tightened EHR blocking logic to prohibit order translation when AD_Status=Unknown and conflict flags are simultaneously active. After these targeted edits, a second walkthrough pass yielded 100% path convergence across all twelve scenarios, with no jurisdiction-rule conflicts and no logical dead-ends. This convergence indicates internal consistency after refinement and does not constitute empirical validation of clinician usability or clinical effectiveness. All mandatory EHR documentation fields remained completable within the framework's structure throughout both walkthrough passes.**

**Ethics Consultation Triggers: Nine of twelve scenarios (75%) appropriately triggered HEC consultation per the framework's mandatory and discretionary rules. The three scenarios without HEC involvement (S2, S5, S11) were either emergency overrides (S2, S11) where the AD search completed post-stabilization with no subsequent conflict, or cases where the Latest-in-Time rule unambiguously resolved the conflict without requiring deliberation (S5 after applicability refinement). In the two emergency scenarios (S2, S11), the Layer 0 override mechanism functioned appropriately—stabilizing the patient immediately while preserving the subsequent decision pathway—without compromising patient autonomy once capacity or decisional documentation could be assessed.**

**Consensus Achievement: All twelve scenarios reached a legally defensible and ethically reasoned resolution. Importantly, "consensus" does not mean all parties were emotionally satisfied, but rather that the framework produced a clear decision grounded in applicable law (Layers 1-3) with documented justification, and that conflicts were either resolved through HEC mediation or clearly adjudicated by a controlling legal authority (e.g., Layer 1 AD in KR scenarios). Critically, no violations of jurisdiction-specific legal requirements occurred across any scenario, demonstrating the toggle mechanism's effectiveness in maintaining legal compliance across KR, CN-Local, and CN-No-Rule environments.**

**Jurisdictional Robustness: The framework's toggle mechanism operated successfully across all three legal environments. In KR scenarios, the statutory form verification (Form No. 6, Form No. 13) and national registry queries functioned as designed. In CN-Local scenarios, the local enabling rules (e.g., Shenzhen medical record annotation requirements) provided clear pathways for AD enforceability and proxy appointment. In CN-No-Rule scenarios, the mandatory HEC pathway prevented unilateral surrogate decisions and ensured deliberative substituted-judgment processes grounded in the patient's values.**

**Supplementary Table S5. Applicability Checklist (Layer 1 directive scope & applicability parsing)**

| **Checklist Item** | **Mandatory question (answer Yes / No / Unclear)** | **Operational interpretation (within APDM Layer 1 workflow)** | **If Unclear / Contested** |
| --- | --- | --- | --- |
| AC-1 | Does the directive explicitly mention the present intervention or condition? | “Yes” supports applicability for Layer 1; “No” indicates the directive does not clearly govern the present decision and should not be treated as a controlling Layer 1 instruction. | Treat as an applicability dispute → mandatory ethics consultation per Layer 1 triggers; document rationale. |
| AC-2 | Does the directive contain conditional language (e.g., “if terminal,” “if no hope of recovery”)? | “Yes” requires AC-3 to be satisfied before treating the directive as applicable to the present condition. | Treat as an applicability dispute → mandatory ethics consultation; document the conditional clause and its interpretation. |
| AC-3 | Is the anticipated condition currently present? | “Yes” supports applicability when the directive is conditional; “No” indicates the directive is not presently operative under Layer 1. | Treat as an applicability dispute → mandatory ethics consultation; document current-condition assessment. |

**Notes:**

This checklist operationalizes directive applicability parsing within APDM Layer 1 and is applied when a directive is identified during DtS and may be relevant to the present clinical decision.

The checklist comprises three mandatory questions (AC-1 to AC-3) and was introduced during iterative refinement to resolve observed walkthrough divergences.

If any item is marked Unclear/Contested, the case is treated as an applicability dispute and routed to mandatory ethics consultation under the Layer 1 trigger logic, with documented rationale.

Worked examples used in refinement include: whether a directive declining “mechanical ventilation” encompasses short-term post-operative ventilation, and whether “no prolonged suffering” encompasses PEG placement.

A separate Local-Enabled? guard is used in China modes to prevent misapplication of local enabling logic in non-enabling jurisdictions (see Supplementary Table S6).

The Applicability Checklist is a routing tool: an “Unclear/Contested” result does not authorize withdrawal; it triggers ethics escalation and, where appropriate, a time-limited trial with explicit withdrawal criteria.

**Supplementary Table S6. If–Then Decision Rules (APDM + DtS implementation specification)**

| **Step** | **IF (trigger/condition)** | **THEN (action)** | **Mode notes (KR / CN-Local / CN-No-Rule)** | **Required documentation / EHR fields (examples)** |
| --- | --- | --- | --- | --- |
| R-0 | A case requires bedside EOL decision-making while patient lacks capacity or conflict exists | Activate the framework and select the jurisdictional toggle | Toggle logic follows KR / CN-Local / CN-No-Rule as defined in the main text | Record active mode (Jurisdiction) (example field used in DtS timeline). |
| R-1 | Patient is ED and stabilized | Initiate DtS Initial Search within the ED window | ED window is 30–60 min post-stabilization | Initial_Search_Timestamp (Supplementary Table S2). |
| R-2 | Patient is ICU/Ward admission | Initiate DtS Comprehensive Search within ICU/Ward window | ICU/Ward window is within 2 hours of admission | Comprehensive_Search_Timestamp (Supplementary Table S2). |
| R-3 | DtS search is initiated | Perform Registry Query as part of the search | KR mode mandates registry queries; if no registry exists, document “N” with justification | Registry_Query_Completed (Y/N; Timestamp); “N requires justification” (Supplementary Table S2). |
| R-4 | DtS search is initiated | Perform Family/Proxy Inquiry as part of the search | Applies across modes (proxy/kin information affects Layer selection and dispute triggers) | Family_Inquiry_Completed (Y/N; Persons_Asked); “N requires justification” (Supplementary Table S2). |
| R-5 | Any document is discovered (AD or proxy evidence) | Perform Document Verification and update AD fields | Verifiability depends on active mode (registry/statutory form vs medical-record annotation vs evidence-graded) | AD_Status (Found/NotFound/Unknown); AD_Type (Supplementary Table S2). |
| R-6 | Emergency, time-critical action required before search completion | Apply Emergency Override: stabilize first; complete DtS and decision pathway post-stabilization | Emergency override is explicitly allowed with post-stabilization completion | Document override + post-stabilization completion (reflected in scenarios S2/S11 and Extended Findings). |
| R-7 | Conflict or ambiguity is present at any step | Trigger Ethics Consult immediately | Applies across modes; CN-No-Rule additionally requires ethics escalation before withdrawal decisions in high-risk conflicts | Ethics_Consult_Triggered (Y/N; Ticket_No); auto-trigger “Y” if any key step incomplete or conflict noted. |
| R-8 | An AD is found and is potentially applicable | Apply Applicability Checklist (S5) (AC-1 to AC-3) | Checklist is embedded in Layer 1 workflow (Extended Findings) | Record applicability rationale (as part of documented justification) and ethics trigger if contested. |
| R-9 | Local-Enabled? guard check for China modes | IF CN setting lacks enabling local rule → do not apply local-enabling “controlling” logic | Prevents misapplication of statutory/local rules in non-enabling jurisdictions | Guard explicitly introduced in refinement; document active mode determination. |
| R-10 | Layer selection (APDM hierarchy) | Apply APDM: Layer 1 (Verifiable AD) → else Layer 2 (Appointed Proxy) → else Layer 3 (Default Kin) | Layer definitions and triggers are specified in the manuscript; CN-No-Rule treats ADs as primary evidence and uses ethics pathway before withdrawal in high-risk conflicts | Record decisional basis (example field: Legal_Basis=Layer1_AD) and active mode (Jurisdiction). |
| R-11 | Layer 1 criteria satisfied (verifiable + applicable + not revoked; latest-in-time where multiple) | Treat directive as governing within the active mode; document family objection as non-decisional where applicable | In KR/CN-Local, family objection is documented but non-decisional when Layer 1 criteria are met; in CN-No-Rule, directive functions as evidence and high-risk withdrawal requires ethics review | Record verification steps and ethics triggers if contested; (example: AD_Explained_to_Family=Y). |
| R-12 | Layer 2 activated (no controlling directive; valid proxy exists) | Proxy exercises substituted judgment; ethics consult mandatory if proxy–family conflict or value-divergence indicators exist | Proxy conflict triggers are explicitly defined in main text | Document proxy basis and ethics consult if triggered (Illustrative S6 logic). |
| R-13 | Layer 3 activated (no AD or proxy) | Default kin acts as gap-filling surrogate; “disagreement” triggers ethics consultation | KR requires intra-tier consensus; China follows guardianship principles/local policy; disagreement types defined in main text | Document conflict type and ethics consult outcome (Illustrative S12; Supplementary S4). |
| R-14 | +1 Order translation (after Layer 1–3 or emergency override) | Translate decision into executable orders (e.g., POLST/DNR) with auditable documentation | +1 layer requires mandatory fields and audit trail | Example output: POLST_Issued=Y; DNI order issued in DtS timeline. |
| R-15 | EHR gating (blocking rule) | IF AD_Status=Unknown AND conflict flags are active → prohibit order translation until resolved | Blocking logic was tightened during refinement | “Prohibit order translation when AD_Status=Unknown and conflict flags are simultaneously active.” |
| R-16 | Conflict-resolution rules apply during translation | Apply: (i) Latest-in-Time precedence; (ii) clear directive prevails over proxy interpretation; (iii) emergency override with post-stabilization completion | These three rules are explicitly stated in the +1 execution description | Record rationale and timestamps/sign-offs as part of the auditable record. |

Notes:

This table provides an implementation-ready if–then rule set that operationalizes the DtS protocol, jurisdictional toggles (KR/CN-Local/CN-No-Rule), APDM layer selection (Layers 1–3), and +1 order translation.

Time windows and documentation fields align with the DtS specification (e.g., ED post-stabilization window and ICU/ward admission window) and the auditable EHR field structure presented elsewhere in the supplementary materials.

CN-No-Rule is implemented as value-evidence handling (not automatically controlling), with mandatory ethics consultation before withdrawal/limitation decisions in high-risk conflict, consistent with the manuscript’s toggle definitions.

The Local-Enabled? guard ensures that local enabling logic (e.g., medical-record annotation regimes) is applied only where such enabling rules exist and are selected by the toggle.

The EHR gating/blocking rule prevents order translation when key uncertainty and conflict flags are simultaneously active, ensuring that high-risk decisions are not executed without resolution and documentation.


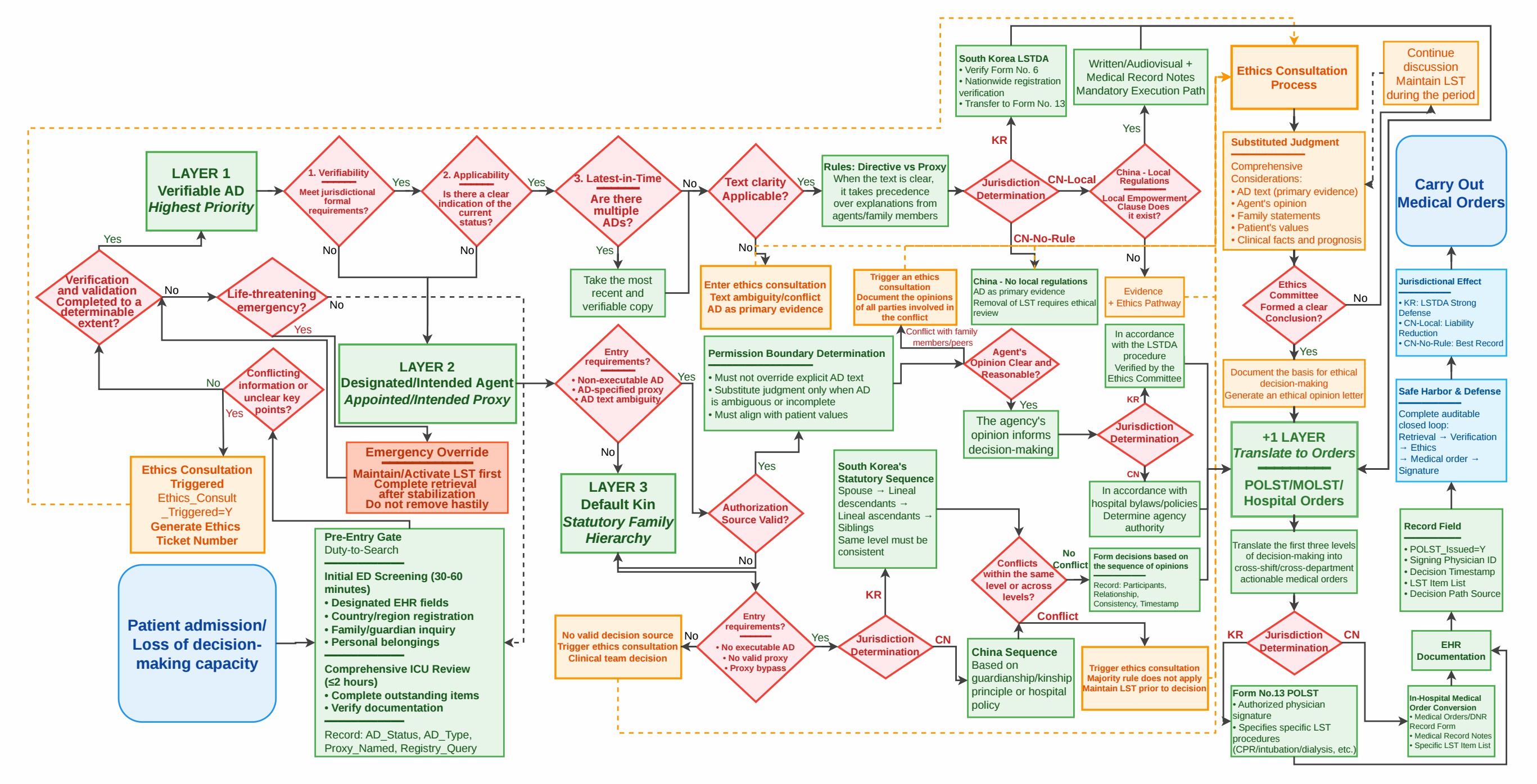


**Supplementary Figure S1**

**Figure 1. “3+1 Priority” & Bedside Conflict Workflow (ED/ICU/Ward). Legend includes Latest-in-Time, Directive-vs-Proxy, and Emergency Override; CN branches show Local-enabled vs No-Rule paths.**
